# Association between breakfast consumption frequency and handgrip strength and standing long jump: a systematic review and meta-analysis

**DOI:** 10.3389/fpsyg.2024.1451799

**Published:** 2024-12-13

**Authors:** Zhongyu Ren, Xiaoping Zhang, Yanqing Wei, Shuai Liu, Bing Cao, Hejin Wang

**Affiliations:** ^1^College of Physical Education, Key Laboratory of Physical Fitness Evaluation, Motor Function Monitoring, General Administration of Sport of China, Southwest University, Chongqing, China; ^2^School of Physical Education, Chinese Center of Exercise Epidemiology, Northeast Normal University, Changchun, China; ^3^Key Laboratory of Cognition and Personality, Faculty of Psychology, Ministry of Education, Southwest University, Chongqing, China; ^4^Sports Faculty Department, Liaoning University, Shenyang, China

**Keywords:** breakfast consumption frequency, grip strength, standing long jump, meta-analysis, review

## Abstract

**Background and aims:**

Over the past decade, numerous studies investigating the relationship between breakfast consumption frequency and handgrip strength and standing long jump have produced conflicting evidence, leading to uncertainty regarding this association. This study aimed to provide further clarity on the relationship between breakfast consumption frequency and handgrip strength and standing long jump.

**Methods:**

A comprehensive search of the literature up to September 15, 2023, was conducted on Web of Science, PubMed, Scopus, MEDLINE, and CNKI. Six studies related to grip strength and three studies related to standing long jump performance were included in the meta-analysis. For studies defining breakfast consumption frequency as an ordinal variable, the effect sizes of the lowest and highest frequency groups were analyzed.

**Results:**

No significant differences in handgrip strength were found between the highest and lowest breakfast consumption groups. Furthermore, unlike in men, women with the highest frequency of breakfast consumption showed significantly higher handgrip strength levels than women with the lowest frequency of breakfast consumption. No significant positive associations were found between breakfast intake frequency and standing long jump levels.

**Conclusion:**

These results suggest that while regular breakfast consumption may enhance handgrip strength in women, it does not influence standing long jump performance in both sexes.

**Systematic review registration:**

https://www.crd.york.ac.uk/PROSPERO/, CRD42024547903

## 1 Introduction

Sustaining optimal muscle strength is crucial for public health (Zhu et al., 2020). Increasing evidence suggests that superior muscle strength in adolescence, adulthood, and old age can help prevent various chronic non-communicable diseases, such as type 2 diabetes(Fraser et al., 2021), hypertension (Oliveira et al., 2023), and cardiovascular diseases (Peralta et al., 2023). Unfortunately, muscle strength levels as reflected by grip strength or standing long jump have decreased in both the adolescent and general population (Masanovic et al., 2020; Li et al., 2023; Fifth National Physical Fitness Monitoring Communiqué, 2022; Kidokoro et al., 2020; Emeljanovas et al., 2020; Kasović et al., 2021). For example, a systematic review indicated that a substantial majority of studies showed a constant decline in muscle strength (Masanovic et al., 2020). Further, a long-term, large-scale study in China found a significant decline in standing long jump levels over time in a representative sample of adolescents aged 7–18 years between 1985 and 2019 (Li et al., 2023). The muscular strength of adults men in 2020 was found to be significantly lower by 1.9% compared to that of adults men in 1984, however, a significant decline was not observed in women’s muscular strength during the same period (Fifth National Physical Fitness Monitoring Communiqué, 2022). Meanwhile, a study in Japan revealed a significant decline in grip strength among college students over the past few decades. Compared to Japanese college students in 1984, the grip strength of Japanese college students in 2016 was significantly lower by 8.1 kg (Kidokoro et al., 2020). Similar patterns of decline in muscle strength were observed in both Asian and Western populations. For instance, Lithuanian children and adolescents (Emeljanovas et al., 2020), as well as Croatian children and adolescents (Kasović et al., 2021) showed a declining trend in grip strength or standing long jump over time. These findings highlight the importance of investigating preventive factors that affect muscle strength, as they can provide valuable insights into the overall health of the population.

Results from epidemiological studies have shown that specific foods or nutrients, including lycopene (Sahni et al., 2021), dairy products(Miyazaki et al., 2023), vegetables and fruits (Yokoyama et al., 2021), fatty fish (Khaing et al., 2024; Xu et al., 2022), nuts (Jun and Shin, 2024), and coffee (Ferreira et al., 2022) are associated with higher muscle strength whereas ultra-processed food intake is associated with lower muscle strength (Zhang et al., 2022). There is also evidence that dietary patterns [e.g., balanced and rice-meat dietary patterns (Liu et al., 2021), plant-based diet (Liu et al., 2021), and plant-poultry-based diet (Shahinfar et al., 2020)] are associated with higher muscle strength. However, researchers have recently focused on breakfast, as breakfast intake can affect an individual’s total-day diet quality (Cuadrado-Soto et al., 2020). Breakfast is typically defined as the first meal consumed within 2 h of waking up, ideally before 10 am. It is considered an important meal, contributing approximately 20–35% of the total daily energy consumption (Jabri et al., 2021). Prior study has demonstrated that subjects who habitually skip breakfast exhibit elevated levels of chronic inflammation (Zhu et al., 2021). The evidence indicates that an increase in inflammatory cytokines, including CRP, IL-6, and TNF-*α*, is associated with a reduction in muscle mass and strength. The decline in inflammatory cytokines has been observed to result in a reduction in anabolic factors, such as insulin and insulin-like growth factor (IGF-1), which can subsequently lead to a down-regulation of muscle protein synthesis (Cervo et al., 2020). It can be reasonably speculated, therefore, that habitual skipping of breakfast may have a deleterious effect on the decline of skeletal muscle strength through the process of elevated systemic inflammation.

Conversely, consuming a regular breakfast is associated with numerous physical and mental health benefits. From a physical perspective, it has been linked to a reduced risk of obesity (Ma et al., 2020), hypertension (Li et al., 2022) and cardiovascular disease (Chen et al., 2020). In terms of mental health, breakfast consumption frequency has been associated with a lower risk of depression (Zahedi et al., 2022) and anxiety (Zahedi et al., 2022). Over the past decade, several studies have investigated the relationship between breakfast consumption frequency and physical function, particularly skeletal muscle strength (Huang et al., 2014; Cui et al., 2018; Ding et al., 2020; Jang et al., 2020; Cuenca-García et al., 2014; Zaqout et al., 2016; Annan et al., 2020). However, there is conflicting evidence regarding the association between breakfast consumption frequency and grip strength. While some studies (Huang et al., 2014; Cui et al., 2018; Ding et al., 2020) have reported a positive association, others (Jang et al., 2020; Cuenca-García et al., 2014; Zaqout et al., 2016; Annan et al., 2020) have not.

To date, existing studies from various countries report a prevalence of irregular breakfast consumption ranging from approximately 8–33% (Yokoro et al., 2021). Breakfast habits, as a modifiable dietary behavior, have significant public health implications in preventing the decline of muscle strength (Khaing et al., 2024). Therefore, our study aimed to systematically review whether a regular breakfast consumption frequency (≥six times/week) is significantly associated with higher skeletal muscle strength in the upper and lower limbs assessed through grip strength and standing long jump.

## 2 Materials and methods

Following the guidelines set by the Preferred Reporting Items for Systematic Reviews and Meta-Analyses (PRISMA), this study has been registered at https://www.crd.york.ac.uk/PROSPERO/ (Registration number: CRD42024547903).

### 2.1 Literature search

A thorough literature search of five databases, including Web of Science, PubMed, Scopus, Medline, and the China National Knowledge Infrastructure (CNKI), was conducted from inception to September 15, 2023 to identify relevant studies that examined the relationship between the frequency of breakfast consumption and handgrip strength and standing long jump. The specific search terms are listed in the Supplementary material. To search for relevant literature, the following search terms were used: (“muscle strength” OR “handgrip strength” OR “grip strength” OR “fitness” OR “physical fitness”) AND (“breakfast frequency” OR “breakfast” OR “breakfast skipping” OR “breakfast omission”).

### 2.2 The inclusion and exclusion criteria

The inclusion criteria were as follows: (1) studies focusing on human populations; (2) those with observational study designs; (3) those that used breakfast consumption as the exposure variable; and (4) those that used handgrip strength and standing long jump as outcome variables. Studies were excluded if they were animal experiments or review articles, did not examine the relationship between breakfast consumption frequency and handgrip strength and standing long jump, or were published in languages other than English or Chinese.

### 2.3 Data extraction and quality assessment

Two independent investigators collected the following information by reviewing each included article using a standardized form: author (publication year), country, study population, sex (sample size), age, exposure variable, exposure assessment, outcome variable, outcome assessment, adjusted confounding factors, mean handgrip strength or standing long jump, and measures of variability [standard deviation, standard error, or 95% confidence interval (95% CI)] for each category of breakfast consumption frequency. The mean handgrip strength or standing long jump with the most appropriate adjustment for confounding factors was extracted. Subgroup analyses were performed based on sex (male and female), adjustment for confounders (yes or no), and assessment tools (Japan and others). The study also extracted the effect estimates for the highest and lowest frequency of breakfast consumption after adjusting for confounding factors. The present study did not employ any automated tools during the process of data collection.

The quality of the included studies in this study was assessed using the Newcastle-Ottawa Scale (NOS). The NOS includes three dimensions: participant selection (0–4 points), comparability (0–2 points), and outcome (0–2 points). The scores of all the items within these dimensions were summed to obtain a total quality score. A total score of ≤5 was considered low quality, 6–7 was considered intermediate quality, and 8–9 was considered high quality.

### 2.4 Statistical analysis

The meta-analysis aimed to assess the association between breakfast consumption frequency and handgrip strength or standing long jump was performed using Stata 17.0. Due to different grip strength measurement tools, we used standardized mean difference (SMD) to express the impact between the highest and lowest categories of breakfast consumption frequency. Conversely, the mean difference (MD) was used to evaluate the standing long jump between the categories with the highest and lowest breakfast consumption frequency.

I^2^ statistics was used to examine the heterogeneity of effect estimates among different studies. As per prior studies, *I*^2^ values between 25 and 49% were considered low, 50–74% were considered moderate, and 75–100% were considered high heterogeneity. When there is a high degree of heterogeneity (*I*^2^ > 50%), the random-effect model was employed for meta-analysis. The subgroup analysis was conducted based on geographical region (Asian, or Non-Asian), sex (male or female), age (<18 or ≥ 18 years), measurement tools (same or different), and adjustment for confounders (yes or no). Sensitivity analysis was performed to assess the impact of including or excluding any single study on the heterogeneity of integrated studies. Publication bias was assessed using the funnel plot with Egger’s test. For all two-sided tests, statistical significance was set at <0.05.

## 3 Results

### 3.1 Literature search and screening

**Figure 1:**
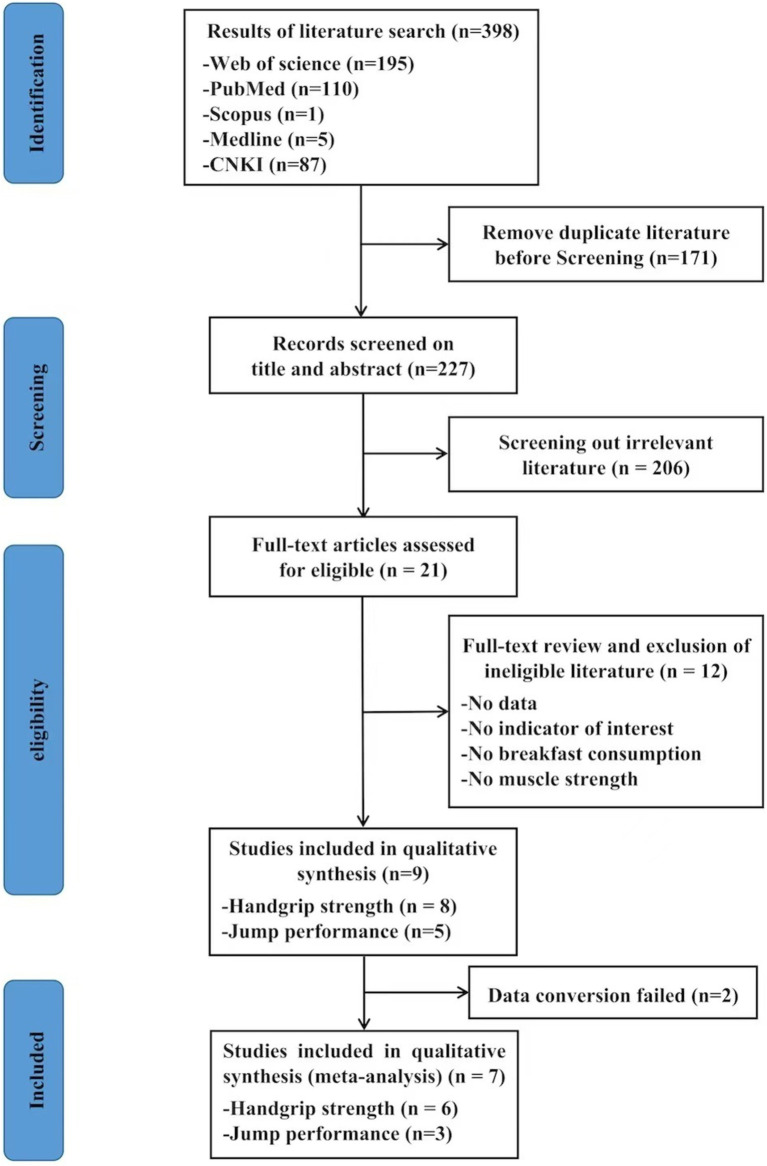
Flow diagram of study selection.

Figure 1 provides a summary of the literature search and screening process. Initially, a total of 398 relevant papers were identified: 195 from Web of Science, 110 from PubMed, 1 from Scopus, 5 from Medline, and 87 from CNKI. Following the exclusion of duplicate based on the titles and abstracts of each article (*N* = 171), we identified 21 eligible articles. Based on the inclusion criteria, only nine full-text articles were deemed eligible; seven of them were included in the meta-analysis.

### 3.2 Basic characteristics of the included overall studies

Among all nine studies (Huang et al., 2014; Cui et al., 2018; Ding et al., 2020; Jang et al., 2020; Cuenca-García et al., 2014; Zaqout et al., 2016; Annan et al., 2020; Gioxari et al., 2023; Hu et al., 2020) that adopted cross-sectional study designs, eight examined the association between breakfast consumption frequency and handgrip strength (Huang et al., 2014; Cui et al., 2018; Ding et al., 2020; Jang et al., 2020; Cuenca-García et al., 2014; Zaqout et al., 2016; Annan et al., 2020; Gioxari et al., 2023), while five examined the association between breakfast consumption frequency and standing long jump (Jang et al., 2020; Cuenca-García et al., 2014; Zaqout et al., 2016; Annan et al., 2020; Hu et al., 2020). These studies reported the mean handgrip strength (with SD, SE, or 95% CI) for each category of breakfast consumption frequency. The studies were conducted in various countries: China (*n* = 3) (Cui et al., 2018; Ding et al., 2020; Hu et al., 2020), Japan (*n* = 1) (Huang et al., 2014), Korea (*n* = 1) (Jang et al., 2020), Ghana (*n* = 1) (Annan et al., 2020), Greece (*n* = 1) (Gioxari et al., 2023), and European countries (*n* = 2) (Cuenca-García et al., 2014; Zaqout et al., 2016). These studies included 25,819 participants, whose age ranges from 6 to 83 years old. Handgrip strength was measured using a hand dynamometer, and breakfast consumption frequency was assessed through self-reported 24-h recalls (Cuenca-García et al., 2014), a brief self-administered dietary history questionnaire (Huang et al., 2014), and other self-reported methods (Cui et al., 2018; Ding et al., 2020; Jang et al., 2020; Zaqout et al., 2016; Annan et al., 2020; Gioxari et al., 2023; Hu et al., 2020). In the nine studies, five included both sexes (Ding et al., 2020; Zaqout et al., 2016; Annan et al., 2020; Gioxari et al., 2023; Hu et al., 2020), while four studies analyzed the association separately for males and females (Huang et al., 2014; Cui et al., 2018; Jang et al., 2020; Cuenca-García et al., 2014). Additionally, Huang et al. (2014) recruited employees who worked in the study region, while Cui et al. (2018) and Ding et al. (2020) recruited regional college students and adults, respectively. The remaining six studies included representative samples of children, adolescents, adults, and older adults (Jang et al., 2020; Cuenca-García et al., 2014; Zaqout et al., 2016; Annan et al., 2020; Gioxari et al., 2023; Hu et al., 2020).

The categorization of breakfast consumption frequency varied across different studies. Chinese population-based studies categorized breakfast consumption frequency into three groups: ≤1 time/week, 2–5 times/week, and ≥ 6 times/week (Cui et al., 2018; Ding et al., 2020; Hu et al., 2020). One Japanese study classified breakfast consumption frequency as ≤2 times/week, 3–5 times/week, and ≥ 6 times/week (Huang et al., 2014). One Korea study classified breakfast consumption frequency as skipping breakfast every day, irregular breakfast with snack, irregular breakfast meal, and breakfast meal everyday (Jang et al., 2020). One Greece study classified breakfast consumption frequency as daily, most of the days, sometimes, and no (Gioxari et al., 2023). The remaining three studies did not specify the number of days per week for the categorization of breakfast consumption frequency (Cuenca-García et al., 2014; Zaqout et al., 2016; Annan et al., 2020). Regarding the measurement of handgrip strength, all included studies used a dynamometer. Among them, a Takei dynamometer was used in three studies (Huang et al., 2014; Cui et al., 2018; Zaqout et al., 2016), a WCS-100 in one study (Ding et al., 2020), a handheld digital dynamometer in one study (Cuenca-García et al., 2014), a Jamar Plus+ Digital Hand Dynamometer in one study (Gioxari et al., 2023), and other types of dynamometers in three studies (Jang et al., 2020; Annan et al., 2020; Hu et al., 2020). The standing long jump test is evaluated based on the distance between the start line and the heel. Detailed information on the basic characteristics of the included studies are shown in Tables 1, 2.

**Table 1:** Characteristics of studies of a meta-analysis investigating the relationship between breakfast consumption frequency and handgrip strength.

Study ID/year	Country	Population	Study design	Sex/total sample size	Age (mean ± SD)	Exposure variable	Exposure assessment	Outcome variable	Outcome assessment	Adjusted confounding factors	Adjusted mean (SD/SE/95%CI)
Cuenca-García et al. (2014)	European cities	Adolescents	Cross-sectional study	2,148	No	Breakfast consumption frequency	Self-reported 24-h recalls	Handgrip strength (kg/kg)	Handheld digital dynamometer (K 5101;Japan)	Center, age, mother’s education, father’s education, and family affluence.	Men: Consumer: 0.5 (0.02) Occasional: 0.5 (0.03) Skipper: 0.5 (0.03) Women: Consumer: 0.6 (0.01) Occasional: 0.6 (0.02) Skipper: 0.6 (0.03)
Huang et al. (2014)	Japan	Adults	Cross-sectional study	Men/723 Women/346	43.8 ± 10.5	Breakfast consumption frequency	Brief self-administered dietary history questionnaire	Handgrip strength (kg)	Handheld digital dynamometer (TKK 5401; Japan)	Age, body mass index, daily energy, protein, vitamin C, vitamin D, a-tocopherol intakes, metabolic syndrome, depression symptoms, and high-sensitivity C-reactive protein, education level, occupation, marital status, sleep duration, toothbrushing, smoking status, drinking frequency, and physical activity.	Men: ≤2 times/week: 41.6 (41.0, 42.2) 3–5 times/week: 41.7 (40.8, 42.6) ≥6 times/week: 42.4 (41.9, 42.9) Women: ≤2 times/week: 25.0 (24.1, 26.0) 3–5 times/week: 25.8 (24.7, 26.9) ≥6 times/week: 25.6 (25.0, 26.3)
Huang et al. (2014)	Japan	Adults	Cross-sectional study	Men/723 Women/346	43.8 ± 10.5	Breakfast consumption frequency	Brief self-administered dietary history questionnaire	Handgrip strength (kg/kg)	Handheld digital dynamometer (TKK 5401; Japan)	Age, body mass index, daily energy, protein, vitamin C, vitamin D, a-tocopherol intakes, metabolic syndrome, depression symptoms, and high-sensitivity C-reactive protein, education level, occupation, marital status, sleep duration, toothbrushing, smoking status, drinking frequency, and physical activity.	Men: ≤2 times/week: 0.613 (0.605, 0.621) 3–5 times/week: 0.616 (0.604, 0.629) ≥6 times/week: 0.625 (0.618, 0.633) Women: ≤2 times/week: 0.479 (0.463, 0495) 3–5 times/week: 0.489 (0.471, 0.508) ≥6 times/week: 0.490 (0.478, 0.501)
Cui et al. (2018)	China	College students	Cross-sectional study	Men/2377 Women/3874	No	Breakfast consumption frequency	Self-reported	Handgrip strength (kg)	Handheld digital dynamometer (TKK 5401; Japan)	Grade, body mass index, race, physical activity, living status, smoking habits, drinking habits, depressive symptoms, and sleep quality.	Men: ≤1 times/week: 41.6 (41.3, 41.9) 2–5 times/week: 42.1 (41.8, 42.4) ≥6 times/week: 42.4 (42.2, 42.7) Women: ≤1 times/week: 25.1 (24.9, 25.4) 2–5 times/week: 25.9 (25.7, 26.2) ≥6 times/week: 26.7 (26.5, 26.9)
Jang et al. (2020)	Korea	Adults	Cross-sectional study	Men/1662 Women/1136	38.14 ± 12.71	Breakfast consumption frequency	Self-reported	Handgrip strength (kg)	Dynamometer	No	Men: skipping breakfast everyday: 43.90 (9.64) irregular breakfast with snack: 43.36 (8.20) irregular breakfast meal: 42.61 (8.88) breakfast meal everyday: 42.44 (9.13) Women: skipping breakfast everyday: 24.15 (5.62) irregular breakfast with snack: 25.01 (5.24) irregular breakfast meal: 25.12 (6.55) breakfast meal everyday: 25.24 (5.72)
Jang et al. (2020)	Korea	Older adults	Cross-sectional study	Men/263 Women/362	72.22 ± 5.25	Breakfast consumption frequency	Self-reported	Handgrip strength (kg)	Dynamometer	No	Men: skipping breakfast everyday: 26.85 (8.88) irregular breakfast with snack: 35.44 (5.20) irregular breakfast meal: 31.83 (7.04) breakfast meal everyday: 32.13 (7.87) Women: skipping breakfast everyday: 21.68 (3.45) irregular breakfast with snack: 20.34 (4.91) irregular breakfast meal: 21.32 (5.36) breakfast meal everyday: 20.34 (5.45)
Annan et al. (2020)	Ghana	Adolescents	Cross-sectional study	Men/213 Women/225	11.1 ± 1.1	Breakfast consumption frequency	Self-reported	Handgrip strength (kg)	Dynamometer	No	Consumer: 4.4 (2.2) Non-consumer: 4.6 (1.8)
Ding et al. (2020)	China	Adults	Cross-sectional study	Men/1171 Women/838	25–65	Breakfast consumption frequency	Self-reported	Handgrip strength (kg)	Handheld digital dynamometer (WCS-100; Shanghai, China)	Age, sex, body mass index, hypertension, diabetes, depressive symptoms, physical activity, educational level, occupation, living status, smoking and drinking habits.	≤1 times/week: 35.2 (34.7, 35.8) 2–5 times/week: 36.0 (35.6, 36.4) ≥6 times/week: 36.9 (36.6, 37.3)
Zaqout et al. (2016)	European cities	Children	Cross-sectional study	4,903	6–11	Breakfast consumption frequency	Self-reported	Handgrip strength (kg)	Dynamometer (TKK 5101 Grip D, Takey)	No	β: −0.002
Gioxari et al. (2023)	Greece	Children	Cross-sectional study	134	6–11	Breakfast consumption frequency	Self-reported	Handgrip strength (kg)	Jamar Plus+ Digital Hand Dynamometer (Patterson Medical, Warrenville, IL, United States)	No	Daily or Most of the Days: 14.5 (5.0) Sometimes/No: 16.2 (3.8)

**Table 2:** Characteristics of studies of a meta-analysis investigating the relationship between breakfast consumption frequency and standing long jump.

Study ID/year	Country	Population	Study design	Sex/total sample size	Age (mean ± SD)	Exposure variable	Exposure assessment	Outcome variable	Outcome assessment	Adjusted confounding factors	Adjusted mean (SD/SE/95%CI)
Jang et al. (2020)	Korea	Adults	Cross-sectional study	Men/1662 Women/1136	38.14 ± 12.71	Breakfast consumption frequency	Self-reported	Standing long jump (cm)	The distance between the start line and the heel	No	Men: skipping breakfast everyday: 200.28 (32.42) irregular breakfast with snack: 193.81 (29.77) irregular breakfast meal: 200.64 (31.12) breakfast meal everyday: 195.15 (31.65) Women: skipping breakfast everyday: 144.09 (26.39) irregular breakfast with snack: 136.03 (28.68) irregular breakfast meal: 142.22 (25.25) breakfast meal everyday: 137.73 (26.38)
Hu et al. (2020)	China	Adolescents	Cross-sectional study	Men/959 Women/890	15.53 ± 1.80	Breakfast consumption frequency	Self-reported	Standing long jump (cm)	The distance between the start line and the heel	No	Men: Breakfast skippers: 223.89 (22.93) Non-breakfast skippers: 226.25 (22.44) Women: Breakfast skippers: 170.27 (16.16) Non-breakfast skippers: 175.61 (17.08)
Zaqout et al. (2016)	European cities	Children	Cross-sectional and follow-up study	Cross-sectional study: 4893 follow-up study: 2,263	6–11	Breakfast consumption frequency	Self-reported	Standing long jump (cm)	The distance between the start line and the heel	Adjustment for moderate-to-vigorous physical activity	Cross-sectional study: β: −0.002 follow-up study: β: −0.019
Annan et al. (2020)	Ghana	Adolescents	Cross-sectional study	Men/213 Women/225	11.1 ± 1.1	Breakfast consumption frequency	Self-reported	Standing long jump (cm)	The distance between the start line and the heel	No	Consumer: 4.6 (2.1) Non-consumer: 5.0 (1.8)
Cuenca-García et al. (2014)	European cities	Adolescents	Cross-sectional study	2,148	No	Breakfast consumption frequency	Self-reported 24-h recalls	Standing long jump (cm)	The distance between the start line and the heel	Center, age, mother’s education, father’s education and family affluence.	Men: Consumer: 187.9 (2.54) Occasional: 183.4 (3.11) Skipper: 185.8 (4.09) Women: Consumer: 147.3 (3.64) Occasional: 144.4 (3.97) Skipper: 147.0 (4.53)

Regarding the outcome measurement, two studies explained the measurement of grip strength and standing long jump (Zahedi et al., 2022; Huang et al., 2014) and indicated that staff need to undergo training. One study conducted a pre-test for staff (Zahedi et al., 2022). Another study (Huang et al., 2014) indicated that six staff received a 3-day training and conducted a 1-day data collection program. The remaining seven articles (Zhu et al., 2021; Cervo et al., 2020; Ma et al., 2020; Li et al., 2022; Chen et al., 2020; Cui et al., 2018; Jang et al., 2020) did not indicate that staff need to undergo training and pre-experiments to measure grip strength and standing long jump.

Regarding the day/conditions of outcome measurement, one study (Jang et al., 2020) indicated that the measurement of physical fitness was after lunchtime at school; another study (Huang et al., 2014) indicated that measurement was conducted in public primary schools but the time of measurement was not introduced; one study (Zahedi et al., 2022) indicated that the standing long jump was conducted on a smooth and hard surface in primary schools, and one study (Cui et al., 2018) indicated that the measurement site was at the hospital. The remaining studies (Zhu et al., 2021; Cervo et al., 2020; Ma et al., 2020; Li et al., 2022; Chen et al., 2020) did not introduce the day/conditions of outcome measurement.

### 3.3 Quality assessment

After assessing the quality of studies using the NOS, one, three, four, and one studies had quality scores of 8, 7, 6, and 5, respectively. Detailed results for quality assessment are presented in Table 3.

**Table 3:** Quality assessment of included studies investigating the relationship between breakfast consumption frequency and handgrip strength and standing long jump.

Cross-sectional studies	Representativeness of the sample	Sample size	Non-respondents	Ascertainment of the exposure	Study control for age	Study control for any additional factor	Assessment of the outcome	Statistical test	Total score of quality
Cuenca-García et al. (2014)	*	*	*	*	*		*	*	7
Huang et al. (2014)	*	*	*	*	*	*	*	*	8
Cui et al. (2018)			*	*	*	*	*	*	6
Jang et al. (2020)	*	*	*	*			*	*	6
Annan et al. (2020)	*	*	*	*			*	*	6
Ding et al. (2020)			*	*	*	*	*	*	6
Zaqout et al. (2016)	*	*	*	*	*		*	*	7
Hu et al. (2020)	*	*	*	*	*		*	*	7
Gioxari et al. (2023)			*	*	*		*	*	5

The studies were classified as having low quality (≤5), intermediate quality (6–7), and high quality (8–9).

### 3.4 Systematic review results

Regarding handgrip strength, there were discrepant findings in the literature. Four studies (Huang et al., 2014; Cui et al., 2018; Ding et al., 2020; Ding et al., 2020) reported a positive association between regular breakfast consumption and higher handgrip strength levels, while four studies (Jang et al., 2020; Cuenca-García et al., 2014; Zaqout et al., 2016; Annan et al., 2020) found no significant association.

Regarding the standing long jump, one study reported a positive association between regular breakfast consumption frequency and higher standing long jump in both adult males and females (Jang et al., 2020). In another study, the results indicate that regular breakfast was positively associated with standing long jump in women but not in men (Hu et al., 2020). There were no significant positive associations found between regular breakfast consumption and higher standing long jump among children (Annan et al., 2020) and adolescents (Cuenca-García et al., 2014). In another study, no significant associations were found between breakfast consumption and standing long jump among children (Zahedi et al., 2022).

### 3.5 Meta-analysis results

#### 3.5.1 Handgrip strength

**Figure 2:**
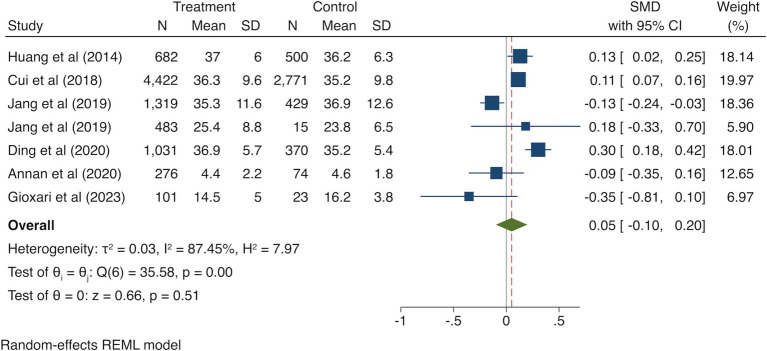
Forest plot of handgrip strength in highest versus lowest category of breakfast consumption.

Six studies (Huang et al., 2014; Cui et al., 2018; Ding et al., 2020; Jang et al., 2020; Cuenca-García et al., 2014; Annan et al., 2020) reported the outcome of handgrip strength of 21,444 individuals. The meta-analysis result indicated no significant association between regular breakfast consumption frequency and higher handgrip strength (SMD = 0.05, 95% CI = −0.10, 0.20; *I*^2^ = 87.45%; *p* = 0.51; Figure 2).

#### 3.5.2 Standing long jump

**Figure 3:**
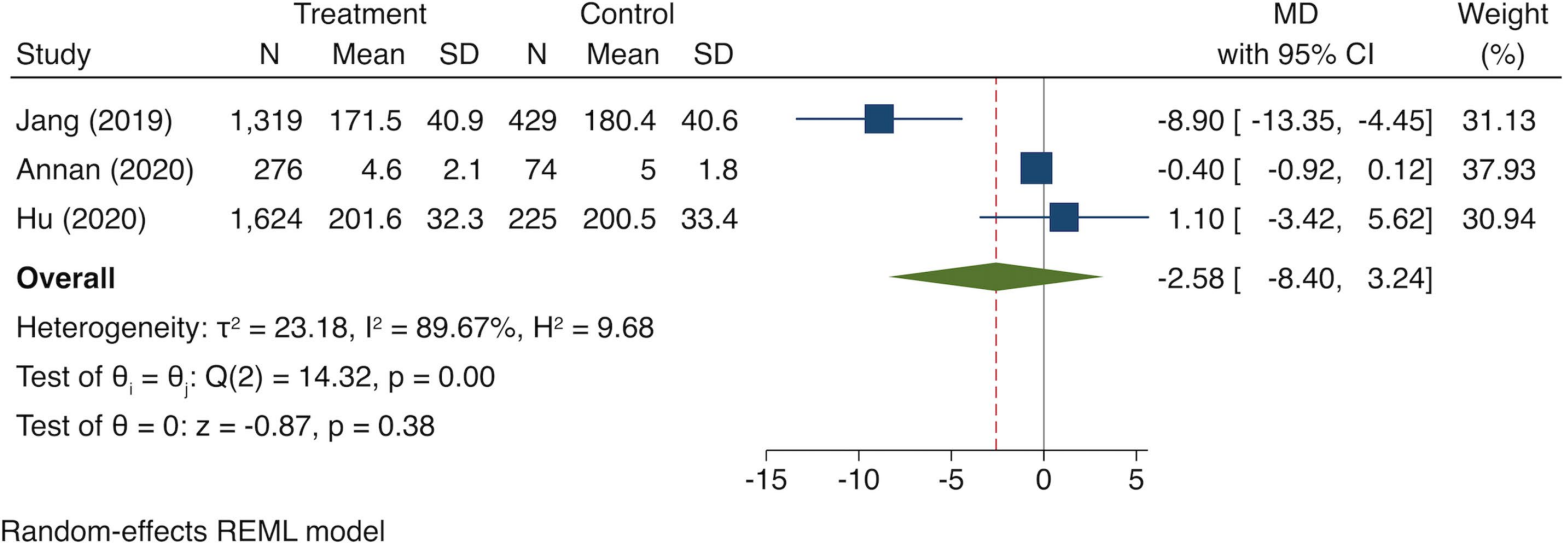
Forest plot of standing long jump in highest versus lowest category of breakfast consumption.

Three studies (Jang et al., 2020; Annan et al., 2020; Hu et al., 2020) reported the outcome of handgrip strength of 5,085 individuals and found that there was no significant difference observed between breakfast consumption frequency and standing long jump (MD = −2.58, 95% CI = −8.40, 3.24; *I*^2^ = 89.67%; *p* = 0.38; Figure 3).

#### 3.5.3 Subgroup analysis

To identify the source of heterogeneity, we conducted a subgroup analysis based on sex, adjustment for significant confounding factors, and measurement tools. In the sex-based subgroup analysis, unlike in men, women with the highest frequency of breakfast consumption showed significantly higher handgrip strength levels than women with the lowest frequency of breakfast consumption (SMD = 0.89, 95% CI = 0.02, 1.77; *I*^2^ = 67%; *p* = 0.04). Furthermore, we found that the association between regular breakfast consumption frequency and higher handgrip strength remained significant, whether adjusting for confounding factors (SMD = 0.17, 95% CI = 0.06, 0.28; *I*^2^ = 76%; *p* = 0.02) or not (SMD = −0.13, 95% CI = −0.22, −0.03; *I*^2^ = 0%; *p* = 0.01). In terms of the measurement tools used for handgrip strength, our subgroup analysis indicated that there is a significant positive association between breakfast consumption frequency and handgrip strength, whether using the same handgrip (SMD = 0.12, 95% CI = 0.07, 0.16; *I*^2^ = 0%; *p* < 0.01) or different handgrip measures (SMD = −0.12, 95% CI = −0.22, −0.02; *I*^2^ = 0%; *p* = 0.02). Detailed results of the subgroup analysis are shown in Table 4.

**Table 4:** Subgroup for the association between breakfast consumption frequency and handgrip strength and standing long jump.

Outcomes	Subgroup	Variables	Number of studies	*I*^2^ (%)	Effect model	Effect size (95% CI)	*p*- value	Weight (%)
Handgrip strength	Geographical region	Asian	5	86	Random model	SMD = 0.11 (−0.03, 0.24)	0.13	83.4
		Non-Asian	2	0	Fixed model	SMD = −0.16 (−0.38, 0.07)	0.17	17.6
	Sex	Male	4	76	Random model		0.57	47.9
		Female	4	67	Random model	SMD = 0.89 (0.02, 1.77)	0.04	52.1
						SMD = 0.31 (−0.75, 1.37)		
	Age	<18 years	2	0	Fixed model	SMD = −0.16 (−0.38, 0.07)	0.17	17.6
		≥18 years	5	86	Random model	SMD = 0.11 (−0.03, 0.24)	0.13	83.4
	Measurement tools	Japan, TKK5401	2	0	Fixed model	SMD = 0.12 (0.07, 0.16)	<0.01	38.08
		Others	5	84	Random model	SMD = −0.00 (−0.23, −0.23)	0.98	61.92
	Mixed factors control	Adjustment	3	76	Random model	SMD = 0.17 (0.06, 0.28)	0.02	84.5
		Non-adjustment						
		Korea (20–64 years)	1	–	–	SMD = −0.13 (−0.24, 0.03)	–	77.87
		Korea (> = 65 years)	1	–	–	SMD = 0.18 (−0.33, 0.70)	–	3.51
		Ghana and Greece	2	0	Fixed model	SMD = −0.16 (−0.38, 0.07)	0.17	18.62
		Unadjusted nutrient intake	6	89	Random model	SMD = 0.03 (−0.15, 0.21)	0.76	100
Standing long jump	Sex	Male	2	83	Random model	MD = −1.33 (−8.68, 6.02)	0.72	49.6
		Female	2	93	Random model	MD = −0.43 (−11.89, 11.03)	0.94	50.4

### 3.6 Sensitivity analysis and publication bias

**Figure 4:**
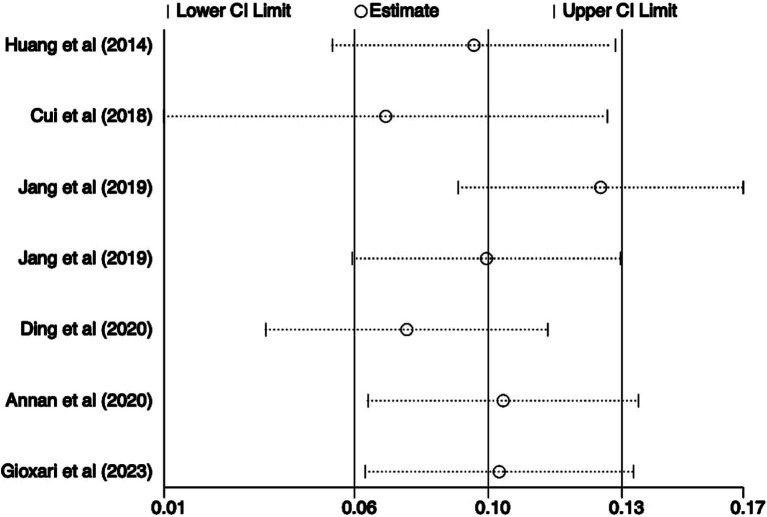
Sensitivity analysis results of included studies for handgrip strength in highest versus lowest category of breakfast consumption.

**Figure 5:**
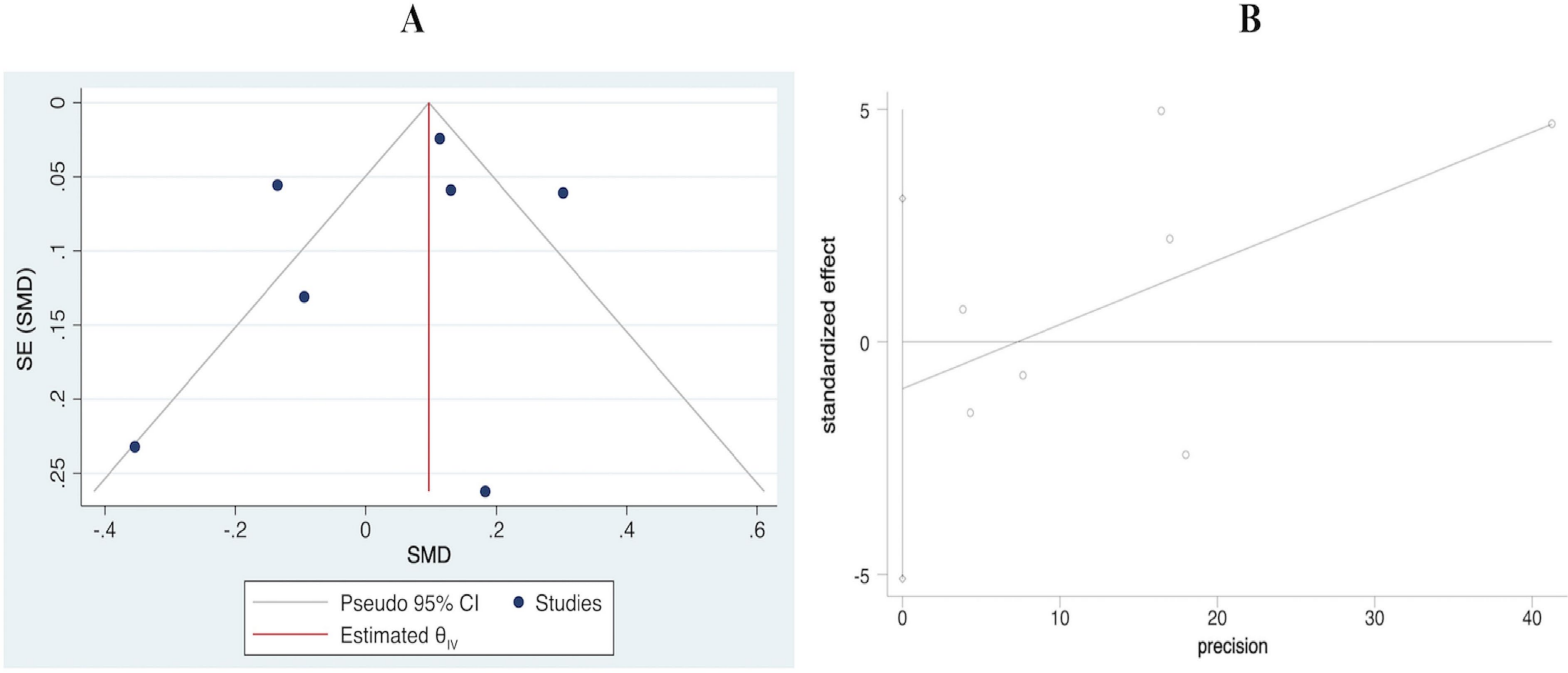
Funnel plot with pseudo 95% confidence limits **(A)** and Egger’s funnel plot **(B)** of handgrip strength in highest versus lowest category of breakfast consumption.

The sensitivity analysis indicated that there was no substantial change in the positive association when each study was omitted one by one (Figure 4). Figure 5 shows that the visualized funnel plot displayed a fairly even distribution of the individual studies within the range of the combined effect size, indicating a modest publication bias.

## 4 Discussion

The systematic review and meta-analysis found that, unlike in men, women with regular breakfast consumption frequency have higher grip strength than women with irregular breakfast consumption frequency. However, there is no significant difference observed in standing long jump between the two groups. To the best of our knowledge, this study is the first of its kind to examine the relationship between breakfast consumption frequency and handgrip strength and standing long jump.

Although the biological mechanisms underlying the effect of breakfast consumption frequency on handgrip strength remain unclear, several potential mechanisms have been explored. A meta-analysis has shown that breakfast consumers have higher intake of minerals, such as calcium and magnesium, as well as vitamins, such as vitamin D, compared to those who skip breakfast (Giménez-Legarre et al., 2020). An intake of vitamin D and calcium may enhance nerve conduction and transmission at the neuromuscular junction, subsequently leading to enhanced contraction of skeletal muscles. Similarly, magnesium plays a crucial role in muscle function and performance. Mg2+ regulates troponin expression by controlling Ca^2+^ concentration gradients and transport (Rayani et al., 2021). Additionally, Mg2+ plays a role in stabilizing protein structures, including actin. Actin, existing as a globular monomer, interacts with ATP and Mg2+, which are essential for the formation of the chemical structure complex required for cross-bridge formation in the sarcomere (Castiglioni et al., 2024). Muscle contraction relies on the relaxation phase, during which Ca2+ is pumped back into the sarcoplasmic reticulum (SR) of the muscle fiber (Castiglioni et al., 2024). This process relies on specific pumping mechanisms that require both ATP and Mg2+ (Castiglioni et al., 2024). Mg2+ serves as an anchor for cofactors like ATP and activates enzyme reactions (Vishnu et al., 2024). Inflammation alteration is likely to explain this association. In a cross-sectional study investigating the impact of skipping breakfast on inflammation alteration, an inverse relationship was found between regular breakfast consumption frequency and chronic inflammation levels (Zhu et al., 2021). The prevention of change in handgrip strength by the mechanisms underlying breakfast consumption frequency may be attributed to the increase in adrenergic activity and blood pressure levels induced by prolonged fasting, which could accelerate inflammatory responses. Based on these findings, we hypothesize that the significant association between regular breakfast consumption and greater handgrip strength may be influenced by the regulation of inflammation levels.

Additionally, sex differences in hand grip strength could explain the significant association between regular breakfast consumption frequency and higher handgrip strength, which was exclusively observed in females compared to males. We explored several potential explanations for the observed sex difference in the present study; in particular, the significant positive association between breakfast frequency and grip strength observed in women but not in men. According to previous studies, among the Asian population included in this study, women had better breakfast habits compared to men, specifically including those rich in protein (Díaz-Torrente and Quintiliano-Scarpelli, 2020). A placebo-controlled, double-blind, randomized controlled trial (Kim et al., 2021) and animal experiments (Aoyama et al., 2021) examined the effect of different timing of protein intake on muscle function and found that regular and adequate breakfast protein intake plays an important role in maintaining muscle strength and mass, with the effect being more pronounced in women. Furthermore, sex differences exist in the diurnal variations of myogenic differentiation 1 (MyoD) expression, a gene involved in muscle differentiation, and these diurnal variations are regulated by clock genes and significantly influenced by dietary intake (Aoyama et al., 2021). Based on these reasons, we speculate that female protein intake during breakfast suppresses the decline in muscle regeneration capacity caused by reduced female hormone secretion and also suppresses the weakened muscle regeneration ability resulting from the same decrease in hormone secretion. Both of these mechanisms are achieved through MyoD.

This study found no significant positive association between breakfast consumption frequency and standing long jump, as compared to grip strength. One possible reason could be that the performance of a standing long jump is dependent not only on lower body muscle strength; an arm swing can also contribute by creating an additional downward force on the body when the major hip and knee extensors are in a better position to exert vertical ground reaction force, thereby improving jumping performance (Zhou et al., 2020).

Previous studies on the impact of upper limb strength on lower limb power have focused on the effects of handheld extra weights while jumping and found that handheld extra weights during the jump process allow for greater muscle activation, resulting in proper muscle contractions. Additionally, dynamic features also showed that as the horizontal translation of the center of mass caused by extra hand weights increases, ground reaction forces also increase (Zhou et al., 2020). This also indicates that the upper limb has a significant effect on lower limb strength.

This study had several limitations. First, the cross-sectional design of the study meant that it could not establish a causal relationship between breakfast consumption frequency and handgrip strength. Second, the subgroup analyses conducted to assess sex, measurement tools, and confounding factors helped reduce heterogeneity, indicating their importance as sources of heterogeneity. However, there may be other unmeasured factors influencing the association. Third, self-reporting of breakfast consumption frequency may be prone to recall errors, potentially affecting the accuracy of the assessment. Fourth, there were other differences between studies, such as the choice of reference and exposure groups, which could have influenced the true association in a positive or negative way. Fifth, while some studies adjusted for important potential confounders, other unknown factors could have confounded the association between breakfast consumption frequency and handgrip strength. Sixth, regular breakfast consumption frequency might also be associated with total daily nutritional intake. For example, two meta-analyses indicated that individuals who had regular breakfast consumption had a higher intake of minerals (iron, calcium, magnesium, potassium, zinc, and iodine) (Giménez-Legarre et al., 2020), carbohydrates, and fiber, as well as a lower fat intake than breakfast-skippers (Giménez-Legarre et al., 2020). According to Cochrane collaboration commandments, ≥10 studies are needed to obtain clear conclusions on publication bias (Nasser, 2020). Egger’s test was used to analyze publication bias of the included studies and the results did not change substantially. However, further research examining the association between breakfast consumption frequency and muscle strength is needed. Seventh, although we have explored the association between breakfast consumption frequency and muscle strength, the type of food and food quantity consumed during breakfast were not specified. However, in previous studies that dairy products were found to be the most common breakfast food (Murakami et al., 2022; Wang et al., 2024), indicating that individuals who regularly consume breakfast tend to have healthier dietary habits, especially those who include protein-rich foods. Eighth, for most studies, the categories of breakfast consumption or breakfast skipping, varied from yes/no to every day/almost every day/3–5 days per week/1–2 days per week/none. However, few studies have provided clear definitions for regular breakfast consumption frequency or skipping breakfast. Finally, a bias derived from methods for collecting data at what timeframe on breakfast consumption frequency could significantly affect the association between regular breakfast consumption frequency and muscle strength. Therefore, these different timeframes make it difficult to draw conclusions about the impact on muscle strength.

## 5 Implications for clinicians and policy makers

In addition to muscle strength, previous meta-analysis have demonstrated positive and negative associations between breakfast consumption and various chronic non-communicable diseases, including depressive symptoms (Zahedi et al., 2022; Ren et al., 2020), cardiovascular disease (Chen et al., 2020; Morze et al., 2020). Although all current results regarding the association between breakfast consumption frequency and muscle strength are widely based on epidemiologic studies and the mechanism underlying the effect of breakfast consumption frequency on muscle strength is uncertain, the primary purpose of these studies was to provide plausible dietary recommendations to prevent the decline of muscle strength, reinforcing the public health message for a rational breakfast consumption frequency. Considering that breakfast is considered an important meal, contributing approximately 20–35% of the total daily energy consumption and daily micronutrients intake (Wang et al., 2024), further research, particularly intervention studies, is required in order to gain a deeper understanding of the quality of food consumed at breakfast, as well as to inform the development of effective policies to promote regular breakfast consumption among the general population, because they are more likely to identify causal relationships. These studies will also provide evidence regarding appropriate and inappropriate breakfast consumption choices that can be used by physicians and patients with decline of muscle strength, as well as information about certain foods of breakfast consumption that could help lower the risk of recurrent decline of muscle strength attacks. This study proposes that in order to provide optimal guidance on evidence-based dietary recommendations, consideration should be given to a comprehensive approach that encompasses not only the prevention of chronic non-communicable diseases, including physical and mental health conditions, but also the potential impact of dietary habits, including the consumption of breakfast, on the risk of muscle strength decline.

## 6 Conclusion

Our systematic review and meta-analysis provide an important contribution to the field by identifying sex differences in the association between breakfast consumption frequency and handgrip strength and standing long jump. Unlike in men, women with regular breakfast consumption frequency have higher grip strength than women with irregular breakfast consumption frequency. However, there is no significant difference observed in standing long jump between males and females. Further studies are needed to examine the association between breakfast consumption frequency in terms of food quantity and specific foods and muscle strength in males and females. Understanding how breakfast characteristics (food quantity and specific foods) influence muscle strength will help design more effective tailored sex-specific breakfast programs that prevent muscle strength decline and improve the overall quality of life. Further meta-analyses of prospective cohort studies or interventional studies evaluating the effect of breakfast consumption on muscle strength are also warranted to determine the causal association. These types of studies will provide more robust evidence regarding the association.

## Data Availability

The original contributions presented in the study are included in the article/Supplementary material, further inquiries can be directed to the corresponding author/s.
