# Supplementary material for: Association between breakfast consumption frequency and handgrip strength and standing long jump: a systematic review and meta-analysis
Source: Front Psychol. 2024 Dec 13;15:1451799. doi: 10.3389/fpsyg.2024.1451799 (PMC11671810; doi:10.3389/fpsyg.2024.1451799)
Supplement: Supplementary file 1 [file Table_1.DOCX]

| **Electronic database** | **Specific search strategies** |
| --- | --- |
| Pubmed | ((muscle strength[MeSH Terms]) OR (muscle strength[Title/Abstract] OR handgrip strength[Title/Abstract] OR grip strength[Title/Abstract] OR fitness[Title/Abstract] OR physical fitness[Title/Abstract])) AND ((breakfast frequency[MeSH Terms]) OR (breakfast frequency[Title/Abstract] OR breakfast*[Title/Abstract] OR breakfast skipping[Title/Abstract] OR breakfast omission[Title/Abstract])) |
| Web of science | ((TI=(muscle strength ORhandgrip strength OR grip strength OR fitness OR physical fitness)) OR AB=(muscle strength ORhandgrip strength OR grip strength OR fitness OR physical fitness)) AND ((TI=(breakfast frequency OR breakfast* OR breakfast skipping OR breakfast omission)) OR AB=(breakfast frequency OR breakfast* OR breakfast skipping OR breakfast omission)) |
| Scopus | TITLE-ABS-KEY ( muscle strength OR handgrip strength OR grip strength OR fitness OR physical fitness ) AND TITLE-ABS-KEY ( breakfast frequency OR breakfast* OR breakfast skipping OR breakfast omission) |
| Medline | ((TI (breakfast frequency OR breakfast* OR breakfast skipping OR breakfast omission) OR AB (breakfast frequency OR breakfast* OR breakfast skipping OR breakfast omission)) AND (TI (breakfast frequency OR breakfast* OR breakfast skipping OR breakfast omission) OR AB (breakfast frequency OR breakfast* OR breakfast skipping OR breakfast omission)) |
| CNKI | TITLE-ABS-KEY ( muscle strength OR handgrip strength OR grip strength OR fitness OR physical fitness ) AND TITLE-ABS-KEY ( breakfast frequency OR breakfast* OR breakfast skipping OR breakfast omission) |

**Table A1.** The specific search strategies in meta-analysis
